# Supplementary material for: Causal interplay between lactose intolerance and gut microbiota: a combined bidirectional Mendelian randomization and in vivo validation study
Source: Front Nutr. 2026 Jun 1;13:1803337. doi: 10.3389/fnut.2026.1803337 (PMC13265576; doi:10.3389/fnut.2026.1803337)
Supplement: Supplementary file 1 [file Data_Sheet_1.zip › supplementary materials/Forward/forest plot/ebi-a-GCST90027844.finngen_R12_E4_LACTONAS.pdf]

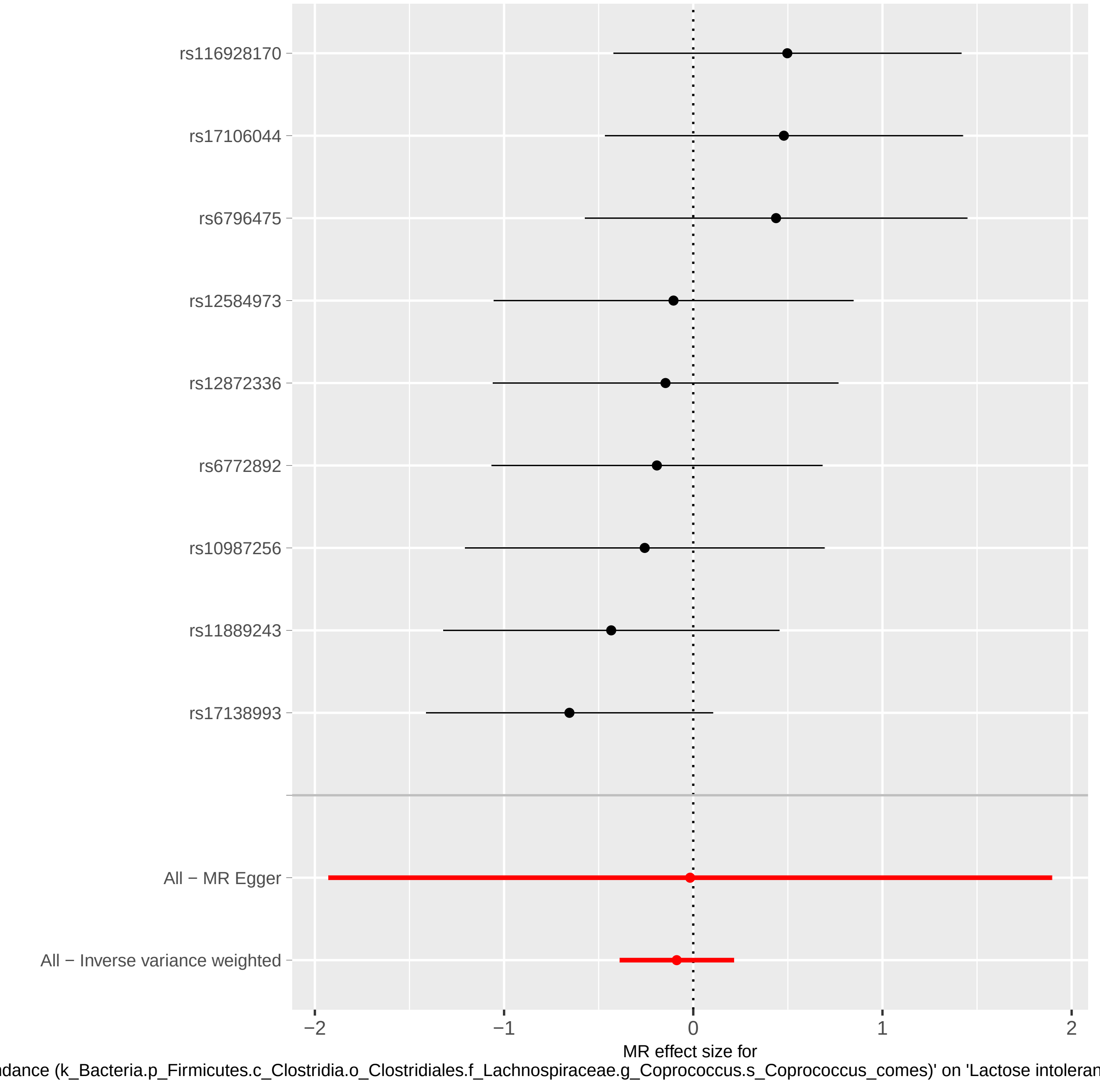
rs116928170
rs17106044
rs6796475
rs12584973
rs12872336
rs6772892
rs10987256
rs11889243
rs17138993
All − MR Egger
All − Inverse variance weighted
−2
−1
0
1
2
MR effect size for
dance (k_Bacteria.p_Firmicutes.c_Clostridia.o_Clostridiales.f_Lachnospiraceae.g_Coprococcus.s_Coprococcus_comes)' on 'Lactose intoleran
